# A New Culture Method for the Detection of Non-Tuberculous Mycobacteria in Water Samples from Heater–Cooler Units and Extracorporeal Membrane Oxygenation Machines

**DOI:** 10.3390/ijerph191710645

**Published:** 2022-08-26

**Authors:** Savina Ditommaso, Monica Giacomuzzi, Gabriele Memoli, Jacopo Garlasco, Antonio Curtoni, Marco Iannaccone, Carla M. Zotti

**Affiliations:** 1 Department of Public Health and Pediatrics, University of Turin, 10126 Torino, Italy; 2 Microbiology and Virology Unit, University Hospital Città della Salute e della Scienza di Torino, 10126 Torino, Italy

**Keywords:** heater–cooler, non-tuberculous mycobacteria, extracorporeal membrane oxygenations, water filtration, culture media

## Abstract

The isolation of non-tuberculous mycobacteria (NTM) from cultures is particularly laborious due to the potential overgrowth of coexisting non-acid fast bacilli. To reduce the overgrowth of these non-mycobacterial organisms, a decontamination step with NaOH or cetylpyridinium chloride is highly recommended before plating the samples on the culture medium. However, due to their toxicity, decontamination solutions tend to decrease NTM recovery from clinical and environmental samples. Here, we tested an alternative method for NTM recovery based on the use of NTM Elite agar, a selective medium that does not require a decontamination step. Using NTM Elite agar, we were able to detect non-tuberculous mycobacteria in 27.7% (30/108) of water samples analyzed. The average time to NTM detection was 18 days, but some strains required longer to grow, perhaps due to the stressful environmental conditions (periodical disinfection of devices). NTM Elite agar’s effectiveness in inhibiting background flora was proven by the isolation of NTM from samples with and without background flora, showing no statistically significant differences in detection rates for different total viable counts of background flora (*p* = 0.4989). In conclusion, our findings indicate that effective NTM recovery from HCU- and ECMO-derived water samples can be achieved via filtration and direct culture of the filters on NTM Elite agar. This simple procedure can speed up laboratory work and provide an improved method, successfully resulting in low contamination and high detection rate, in addition to being less time-consuming. Its sensitivity and lack of a decontamination step make this protocol particularly useful for monitoring the effectiveness of device disinfection in hospital settings, even in the presence of low NTM loads. Reading timeframes should probably be extended to 7 weeks (i.e., well beyond the standard 4 weeks advised by the manufacturer), in order to isolate even the slow-growing mycobacteria. However, an extended incubation period is not necessary for exclusion of *M. chimaera* contamination of the devices, as *M. chimaera* isolation times do not generally exceed 3 weeks.

## 1. Introduction

Evidence to date indicates that heater–cooler units (HCUs) and extracorporeal membrane oxygenation (ECMO) machines used during cardiac surgery can generate infectious aerosols due to the presence of water-borne pathogens such as *Mycobacterium chimaera* [1,2,3,4]. This unwanted effect makes environmental microbiological surveillance of HCU and ECMO water systems an essential step to reduce the risk of healthcare-acquired infections associated with use of HCU and ECMO devices. Ideally, this microbial monitoring should be performed through analytical protocols able to detect even low densities of bacteria.

As reported by several researchers, the isolation of non-tuberculous mycobacteria (NTM) from cultures is particularly laborious due to the potential overgrowth of coexisting non-acid fast bacilli (non-AFB) [5,6,7]. Thus, in order to reduce the overgrowth of these non-mycobacterial organisms, a decontamination step with NaOH or cetylpyridinium chloride (CPC) is highly recommended before plating the samples on the culture medium. However, due to their toxicity, decontamination solutions tend to decrease NTM recovery from clinical and environmental samples on both liquid and solid media. Other factors affecting the recovery of mycobacteria include the type of culture medium and the temperature at which the incubation is performed [8]. 

For microbiological surveillance of HCU and ECMO water systems, the technical document issued by the European Centre for Disease Prevention and Control (ECDC) recommends concentrating water samples by filtration or centrifugation, decontaminating them with NALC-NaOH or CPC and plating on selective media, such as Middlebrook 7H11 or 7H10 agar, or egg-based media, such as Löwenstein–Jensen agar. In addition, culture in liquid medium can be performed using the mycobacteria growth indicator tube (MGIT) 960 system [9]. However, automated cultivation using these special media can become prohibitively expensive when a lab is dealing with a large volume of samples. Another drawback of using these types of media is that, in some cases, they have been shown to limit the growth of environmental bacteria, mainly psychrophilic or thermophilic species [8,10,11]. 

Even though a number of different publications providing useful guidance do exist [7,9,12,13,14,15,16], a standard method for NTM detection has not yet been established. In this regard, variable growth rates, specific growth requirements and different sources of water samples (e.g., treated water systems, natural/surface water, or different devices, e.g., samples from endoscopes or HCUs) are variables that can influence the choice of the most suitable analytical protocol. Furthermore, as mentioned above, pre-treatment steps are often required to limit bacterial and fungal overgrowth, which would otherwise make it difficult to detect precisely the presence of slow-growing mycobacteria species. However, due to their inherent toxicity, pre-treatment procedures may also prevent the detection of certain mycobacteria or reduce the positivity rate and the number of colonies in water samples [5,17,18].

Recent evidence has shown that protocols based on rapidly growing mycobacterium (RGM) media can be highly effective in recovering NTM from potable and non-potable water samples [19]. More specifically, RGM medium alone, without pre-treatment, has been shown to afford higher sensitivity (~91%) and lower overgrowth of contaminating water-borne microorganisms compared to standard media. 

Our microbiological laboratory operates as a regional reference service for microbiological surveillance of all HCUs and ECMOs located in cardiac surgery facilities across the Piedmont region (north-west Italy). Specifically, our lab plays a central role in diagnosing, detecting, identifying, and typing environmental infectious bacterial agents (environmental risks), monitoring the response to epidemics, providing scientific evidence for disease prevention and control, and assisting in the development of regional guidelines on control and risk containment of NTM infection [20,21,22]. Fittingly, we have previously shown that the commercial RGM medium, NTM Elite agar (bioMérieux, Marcy-l’Étoile, France), constitutes an effective alternative method for NTM recovery, as it efficiently inhibits non-AFB without requiring a decontamination step [23]. In this previous study, we used artificially contaminated samples with *M. chimaera* and *Mycobacterium chelonae* to compare recovery efficiency of the membrane filter washing procedure [9] against direct plating of membrane filters on culture media. 

Moreover, we compared the efficacy of NTM Elite agar in inhibiting the growth of aquatic bacteria with that of cetylpyridinium chloride and *N*-acetyl-L-cysteine sodium hydroxide decontamination treatments. We demonstrated that: (i) the washing procedure yielded a low release of both mycobacterium strains from the membrane filters (≈7%), while direct plating of membrane filters led to a 100% cell recovery; (ii) water sample pretreatment with *N*-acetyl-L-cysteine sodium hydroxide, despite achieving complete suppression of non-acid fast bacilli, caused a reduction in mycobacteria growth, while decontamination with cetylpyridinium chloride was found to be ineffective against some non-acid fast organisms. 

Here, we sought to evaluate NTM Elite agar’s performance in recovering NTM from water samples collected from HCU and ECMO devices. Furthermore, we analyzed NTM Elite agar’s selectivity in consideration of interfering bacteria, i.e., total viable counts (TVCs) and *Pseudomonas aeruginosa* in collected samples, by evaluating the relationship between TVC concentration and probability of NTM isolation. 

## 2. Materials and Methods

### 2.1. Microbiological Surveillance in Cardiac Surgery Settings

In this study, we analyzed water samples collected between January 2021 and November 2021 during a microbiological surveillance carried out on 33 devices—26 HCUs manufactured by LivaNova or Maquet Getinge and 7 ECMOs by Maquet Getinge—from nine cardiac surgery facilities and from one sink used by healthcare facility staff to fill water tanks of the hospital’s devices.

### 2.2. Sample Collection

A total of 108 water samples were collected in sterile 1 L plastic bottles, containing 20 mg of sodium thiosulphate. Samples were immediately sent to the laboratory or, if not possible, stored at a controlled temperature (5 ± 3 °C for 12–15 h). All analyses were performed within 24 h of collection. 

### 2.3. Microbiology Workflow

Water samples were tested for NTM, *P. aeruginosa*, and total viable counts (TVCs). TVCs and *P. aeruginosa* are general indicators of water cleanliness and are used as surrogate indicators of decontamination effectiveness. Enumeration of background bacteria in HCU-derived water samples was performed according to the UNI EN ISO 6222 protocol [24]. Briefly, 1 mL of undiluted samples and 1 mL of samples diluted 1:10 in Page’s saline solution were analyzed using the pour-plate method on yeast extract agar. Results were reported as CFU/mL. Samples diluted 1:10 with counts greater than 300 CFU/mL per plate were reported as >3 × 10^3^/mL. When we could not determine the presence of NTM due to fungal growth covering the filter or presence of non-AFB at a concentration >3 × 10^2^ CFU/mL, samples were defined as “unreadable plates”.

*P. aeruginosa* was detected and quantified by culture according to the ISO 16266 method [25], which consists in filtering 250 mL of the water sample using 0.45 μm mixed cellulose esters filters (Millipore, Billerica, MA, USA). Results were recorded as CFU/250 mL.

NTM isolation was performed using the following procedure: 100 mL of each water sample was filtered through mixed cellulose esters filter, and the filter was directly added to a Petri dish containing NTM Elite agar (0.47% Middlebrook 7H9 base, 0.5% glycerol, 1.3% agar, 0.4% yeast extract, 0.2% glucose, 0.5% bovine serum albumin, 0.0056% oleic acid, and 0.0494% of a mix of selective antibiotics and antifungals) (bioMérieux, Marcy-l’Étoile, France). Subsequently, the NTM Elite agar plates were incubated at 30 °C in a sealed plastic bag to prevent dehydration. Starting from the seventh day, cultures were examined weekly for seven weeks. After realizing that mycobacteria from environmental samples needed a longer time to grow, we opted to extend the incubation time from 4 weeks—as advised by the manufacturer—to 8 weeks. 

All colonies growing on NTM Elite agar were confirmed as AFB by Kinyoun stain and then subcultured on Middlebrook 7H11 medium and identified by matrix-assisted laser desorption/ionization-time of flight (MALDI-TOF). NTM were processed according to the MycoEx preparation protocol following the manufacturer’s instructions. Spectra were acquired by Bruker Microflex LT (Bruker Daltonics, Bremen, Germany) MALDI-TOF MS and analyzed by MBT Mycobacteria RUO Library v.4.0 (Bruker Daltonics, Bremen, Germany).

The results from NTM Elite agar analyses were reported as CFU/100 mL.

### 2.4. Quality Control

As satisfactory performance control for this new protocol, we analyzed 6 samples of proficiency test (PT) for NTM recovery in water samples (*Mycobacterium* spp. Water Scheme) distributed by the Food and Environmental Proficiency Testing Unit (FEPTU) of UK Health Security Agency (UK HSA). This microbiology scheme provides samples to laboratories that examine HCU waters for *Mycobacterium* spp. The FEPTU report provides the expected results (i.e., the *Mycobacterium* spp. colony counts in each distributed samples) to participating laboratories [26]. 

### 2.5. Data Analysis

Data were collected and organized using Microsoft Access and Excel 2016 (Microsoft Corporation, Redmond, WA, USA). Hypothesis tests on categorical variables were performed using Fisher’s exact test. Correlation between bacterial contamination (total viable counts), and proportion of detected NTM-positive samples was inspected through LOESS regression. All analytical computation and plotting were carried out using the statistical software R version 4.0.5 (R Foundation for Statistical Computing, Vienna, Austria) [27].

## 3. Results

A total of 102 water samples from 26 HCUs—12 Stockert 3T (LivaNova, Sorin Group, Munich, Germany), 14 HCU40 (Maquet, Getinge Group, Rastatt, Germany)—and from 7 ECMOs (HU35, Maquet, Getinge), were collected and cultured on NTM Elite agar. These 33 devices are regularly disinfected according to the manufacturer’s instructions: 58 of the 102 samples (57%) were collected after disinfection, while 44 (43%) before disinfection. Moreover, 6 water samples were taken from one sink faucet equipped with a point-of-use (POU) filter (this sink was associated with tank-filling of 4 devices—2 HCUs and 2 ECMOs—that turned out to be NTM-positive), bringing the total number of samples to 108.

As shown in Table 1, we successfully isolated NTM from 27.7% (30/108) of all specimens combined. 

Overall, NTM, TVCs, and *P. aeruginosa* results were available from 100 out of 108 samples. The results of this microbiological surveillance are summarized in Table 2.

In order to analyze NTM ELITE agar’s selectivity, we disaggregated the data by TVCs, *P. aeruginosa*, and NTM results. As shown in Table 2, NTM were isolated in 28 samples: 16/58 (27.6%) of samples with background flora vs. 12/42 (28.6%) of those without background flora. Of note, we were able to detect an NTM-positive sample even among specimens with a high bioburden (>3 × 10^3^ CFU/mL). The difference between these proportions was not significant, as Fisher’s exact test analysis indicated that we would not have reached a higher NTM recovery rate in samples without non-AFB (*p* = 0.4989) or *P. aeruginosa* (*p* = 1), indicating that, under our experimental conditions, non-AFB species do not inhibit or mask the growth of NTM colonies on NTM Elite agar.

**Figure 1:**
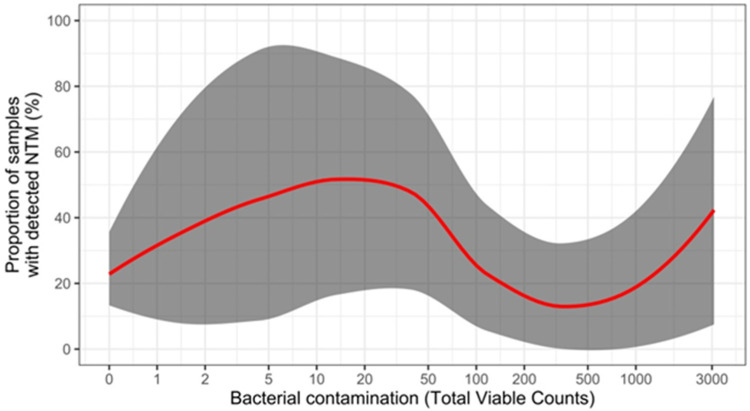
**Proportions of samples with detected NTM according to counts of contaminating bacteria**. The *x*-axis is represented by total viable counts (TVCs) (i.e., contaminating bacteria) and expressed on a logarithmic scale. The red line represents the trend identified by LOESS regression, while the shaded area depicts its corresponding 95% confidence interval (CI).

Locally estimated scatterplot smoothing (LOESS) regression failed to show any particular correlation between the proportion of NTM-positive samples and the presence of different TVCs of contaminating bacteria (Figure 1). Notably, NTM-containing sample proportions exceeded 30% even in samples with TVCs > 1000.

When considering the comparison between TVCs—1 mL of sample analyzed—and the presence of non-AFB on NTM Elite agar—100 mL of sample analyzed—we noticed that 1% of samples yielded non-AFB on NTM Elite agar (unreadable plates) in the absence of overgrowth (Table 3). 

Among samples collected from Stockert 3T HCUs, nine samples did not provide reliable results on NTM Elite agar due to the presence of a large number of non-AFB, eight of them having been collected prior to disinfection and one after HCU disinfection. The results are listed in Table 4. 

NTM prevalence among the devices was 69.2% (18/26) for HCUs—mycobacteria were isolated on more than one sample from some devices—and 28.6% (2/7) for ECMO machines.

A total of 43 isolates belonged to 7 mycobacterial species (*M. chimaera*, *Mycobacterium gordonae*, *Mycobacterium paragordonae*, *M. chelonae*, *Mycobacterium lentiflavum*, *Mycobacterium mucogenicum*, *Mycobacterium frederiksbergense*). *M. chimaera* was isolated only from Stockert 3T HCUs. Specifically, we detected *M. chimaera* in 58.3% (7/12) of Stockert 3T HCUs, including two devices labeled with an *M. chimaera*-free certificate (claimed by the manufacturer), while all Maquet devices—both HCU 40 and ECMO—were negative. Two LivaNova devices were colonized by two different mycobacterium species in the same samples (*M. chimaera*, *M. paragordonae*); one Maquet device was colonized by three different mycobacterium species (*M. lentiflavum*, *M. mucogenicum*, *M. frederiksbergense*).

The time to detection for each NTM species was 12–15 days for *M. chelonae*, 13–18 days for *M. chimaera,* 10–43 days for *M. gordonae,* 17 days for *M. lentiflavum,* 13–18 days for *M. paragordonae,* 10 days for *M. mucogenicum* and 7 days for *M. frederiksbergense.*

**Figure 2:**
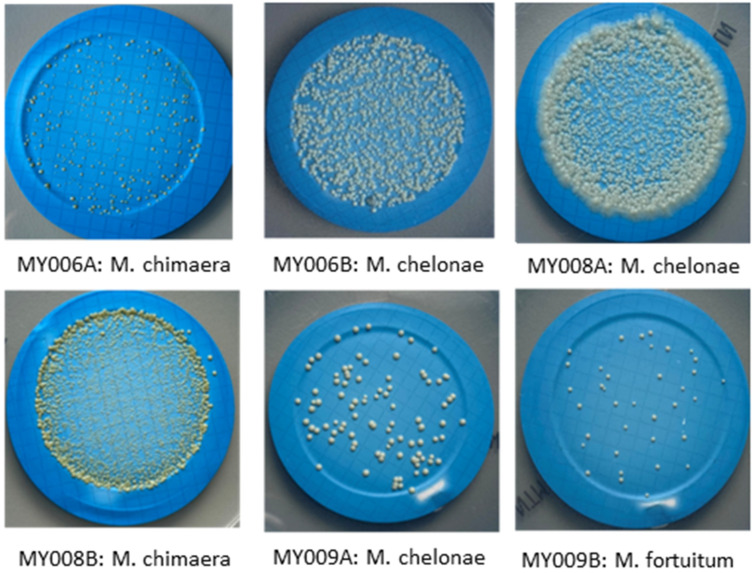
Results of six proficiency samples plated on NTM Elite agar.

All six PT samples obtained from UK HAS FEPTU were correctly classified as NTM-positive. Representative images of our culture results are shown in Figure 2. Table 5 details the expected results declared by the FEPTU report. 

## 4. Discussion

The detection of NTM species in HCU waterlines can be conducted through either molecular approaches [20,21] or culture-based methods, the latter mainly relying on three types of medium: Löwenstein–Jensen, Mycobacteria Growth Indicator Tube (MGIT) 960, and Middlebrook 7H11 [7,13]. Even though culture-based protocols are widely used in clinical microbiology, they generally require long incubation times—six to eight weeks—due to the slow growth rate of mycobacteria. In addition, they are ineffective in detecting viable but non-culturable bacteria under stressful conditions. Moreover, because of their low-analytic sensitivity and high sample contamination rates, these protocols tend to generate low-quality quantitative culture results [28], often biased by the bactericidal activity of the decontamination agents being used [17,18]. Thus, more sensitive detection protocols are urgently needed to perform environmental microbiological surveillance of medical devices.

In the present study, we assessed NTM Elite agar in recovering NTM from HCU- and ECMO-derived water samples. To our knowledge, this is the first study assessing environmental NTM using a protocol based on direct plating of membrane filters on culture medium after filtration. Following this protocol, we were able to detect NTM in 27.7% of water samples analyzed. Specifically, our results show that *M. chimaera* was only isolated from Stockert 3T HCUs, even from devices for which an *M. chimera*-free certificate had been issued by the manufacturer after microbiological analysis of a sample obtained after a deep-disinfection of the device. This is in line with previous findings indicating that *M. chimaera* contamination occurs predominantly in water samples obtained from Stockert 3T LivaNova devices [21]. Therefore, extra infection control precautions should be taken when using these devices, e.g., placing HCUs outside of the operating rooms or venting the exhaust directly to room exhaust [29,30].

The average time to NTM detection was 18 days, but some strains required longer to grow, perhaps due to the stressful environmental conditions (periodical disinfection of devices): for instance, three out of eight *M. gordonae* strains required well beyond the standard 4 weeks to be observed and isolated on the filter.

Overall, NTM were isolated from 27.6% of non-AFB-free samples vs. 28.6% of non-AFB-containing samples (Table 2 and Table 3). This is consistent with the fact that NTM Elite agar contains a combination of selective agents highly effective in inhibiting the vast majority of non-glucose-fermenting Gram-negative bacteria, including *Pseudomonas* species [31], thereby allowing a more efficient NTM recovery. Consistently, our LOESS curve analysis showed no relationship between TVCs concentration and probability of isolation of NTM.

NTM Elite agar’s effectiveness in inhibiting non-AFB contamination is in keeping with a report by Alexander et al. [19], showing that RGM medium generates much fewer samples with non-AFB growth (0.4%) than any other medium tested. In our study, however, we observed overgrowth in 9% of samples, which is somewhat higher than that reported by Alexander and co-workers. This difference could be due to the different processing methods of the samples: direct plating of concentrated samples vs. direct plating of the membrane filter on the NTM Elite agar. In the latter case, the cellulose ester membrane could have interfered on the action of the most important selective agent [32]. However, in NTM Elite agar, 9-chloro-9-[4-(diethylamino) phenyl]-9,10-dihydro-10-phenylacridine hydrochloride [31] prevents the growth of a wide range of bacteria and fungi. Indeed in our previous study [23], we demonstrated that direct plating of filters on agar plate ensures optimal recovery of NTM and a strong inhibition of all tested aquatic bacteria except for *Burkholderia multivorans*. Therefore, the discrepancy with the results of Alexander et al. may be ascribable to the different amounts of sample analyzed in the two studies. While we cultured 100 mL of filtered samples by direct plating of membrane filter, in the study by Alexander et al., only one hundredth of the sample was plated (100 mL of sample were concentrated ten times by filtration, resuspended in 10 mL of sterile distilled water, vortexed, and then 100 μL of the eluate were plated directly onto RGM medium).

A significant shortcoming of our study might be the lack of a direct comparison of the described culture method for NTM to a standard culture method such as concentration by centrifugation [14] or filtration [9] and decontamination followed by culture on 7H11 agar. Indeed, unpublished data, preliminarily obtained on 40 samples analyzed both with direct plating of filters on NTM Elite agar and with the established method [20], have shown a mild concordance between the two methods (Agreement = 75.0%, κ = 0.245), the frequency of NTM isolation being 25.5% with NTM Elite agar (11/40) and 12.5% with the standard protocol (5/40). Furthermore, the previous study [23], conducted on artificial samples, showed that this standard method is actually not very sensitive, due to poor bacteria recovery from the filter and toxicity of reagents used for decontamination.

The results obtained from participation in the PT “*Mycobacterium* spp. Scheme” confirmed the validity of our protocol: we isolated *M. chelonae* from three PT distributions (Figure 2), while 75% of participating laboratories, using MGIT medium, failed to detect *M. chelonae* at levels of approximately 2.2 × 10^2^ and 5.0 × 10^2^ CFUs (distribution number MY006B and MY008A), and all laboratories failed to detect *M. chelonae* at levels 5.8 × 10^1^ CFUs (distribution number MY009A). The root cause for this may lie in the incubation temperature of MGIT: *M. chelonae* grows better at lower temperatures (~30 °C) compared to other mycobacterial species [33,34]. Therefore, samples with low *M. chelonae* loads (e.g., MY009A) can yield a false negative if incubated at temperatures above 30° C. Moreover, according to Kim et al. [35], isolation of *M. gordonae* and *M. paragordonae* strains can be extremely challenging when medium seeding is performed at a temperature above 30 °C. Indeed, *M. gordonae* and *M. paragordonae,* two phylogenetically close species, grow optimally at a temperature of 30 °C—*M. paragordonae* can grow in macrophages at 30 °C, but not at 37 °C [36]. Thus, according to these results, using MGIT medium only for NTM recovery from environmental water samples does not ensure more accurate results simply because specific strains can be missed.

## 5. Conclusions

In conclusion, our findings indicate that effective NTM recovery from HCU- and ECMO-derived water samples can be achieved via filtration and direct culture of the filters on NTM Elite agar at 30° C. This simple procedure can speed up laboratory work and provide an improved method, successfully resulting in low contamination and high detection rate, in addition to being less time-consuming. Its sensitivity (all mycobacteria present in the sample remain trapped on the filter surface) and lack of a decontamination step make this protocol particularly useful for monitoring the effectiveness of device disinfection in hospital settings, even in the presence of low NTM loads. As some samples collected from devices prior to disinfection showed overgrowth, we advise that a lower amount of sample should be filtered and plated, in case higher levels of contamination from other water-borne microorganisms are expected. Moreover, according to our results, we recommend that the reading timeframes should be extended to 7 weeks (i.e., well beyond the standard 4 weeks), in order to isolate even slow-growing mycobacteria. However, an extended incubation period is not necessary for exclusion of *M. chimaera* contamination of the devices, as *M. chimaera* isolation times do not generally exceed 3 weeks.

## Figures and Tables

**Table 1:** NTM isolation by sampling source.

Sampling Source	NTM Positive (%)	NTM Negative (%)	Total
Stockert 3T HCU	14 (31.1)	31 (68.9)	45
Maquet HCU40	12 (27.3)	32 (72.7)	44
Maquet ECMO	2 (15.4)	11(84.6)	13
Sink	2 * (33.3)	4 (66.7)	6
**Total**	30	78	108

* Analyses allowed highlighting damage of the POU filters, which was responsible for contamination of 4 devices in the hospital.

**Table 2:** Comparison of results relative to the three microbiological parameters examined (NTM, TVCs, and *P. aeruginosa*).

	TVCs					*P. aeruginosa*	
	**<1 CFU/Ml ^a^** **(%)**	**1 ≤ CFU/mL ≤ 100 ^b^** **(%)**	**101 ≤ CFU/mL ≤ 1000 ^b^** **(%)**	**1001 ^b^ ≤ CFU/mL ≤3000 ^b^** **(%)**	**>3000 ^a^ CFU/mL** **(%)**	**Positive** **(%)**	**Negative** **(%)**
**NTM positive samples**	16 (27.6)	8 (44.4)	1 (16.7)	2 (40.0)	1 (7.7)	1 (11.1)	27 (29.7)
**NTM negative samples**	41 (70.7)	10 (55.6)	5 (83.3)	3 (60.0)	4 (30.8)	3 (33.3)	60 (65.9)
**Unreadable plates**	1 (1.7)	0 (0.0)	0 (0.0)	0 (0.0)	8 (61.5)	5 (55.6)	4 (4.4)
**Total**	58	18	6	5	13	9	91

TVC: total viable count. **^a^** Both TVCs (22 °C and 36 °C). **^b^** At least one of the two TVCs (22 °C and 36 °C) or both within the limit assigned. (Fisher test for NTM-positive vs. NTM-negative by TVCs: *p* = 0.4989). (Fisher test for NTM-positive vs. NTM-negative by presence of *P. aeruginosa*: *p* = 1).

**Table 3:** Distribution of microbial flora results observed on NTM Elite agar (non-AFB) and TVCs counted on yeast extract agar.

	TVCs					Total
	**<1 CFU/mL ^a^** **(%)**	**1 ≤ CFU/mL ≤ 100 ^b^** **(%)**	**101 ≤ CFU/mL ≤ 1000 ^b^** **(%)**	**1001 ^b^ ≤ CFU/mL ≤3000 ^b^** **(%)**	**>3000 ^a^ CFU/mL** **(%)**	
**Elite NTM negative** **< 1 CFU/100 mL**	41 (70.7)	8 (44.4)	4 (66.7)	2 (40.0)	2 (15.4)	57
**Elite NTM negative** **1–100 CFU/100 mL**	0 (0.0)	2 (11.1)	1 (16.7)	1 (20.0)	2 (15.4)	6
**Elite NTM positive** **< 1 CFU/100 mL**	16 (27.6)	7 (38.9)	1 (16.7)	1 (20.0)	1 (7.7)	26
**Elite NTM positive** **1–50 CFU/100 mL**	0 (0.0)	1 (5.6)	0 (0.0)	1 (20.0)	0 (0.0)	2
**Unreadable plates**	1 (1.7)	0 (0.0)	0 (0.0)	0 (0.0)	8 (61.5)	9
**Total**	58	18	6	5	13	100

TVC: total viable count. **^a^** Both TVCs (22 °C and 36 °C). **^b^** At least one of the two TVCs (22 °C and 36 °C) or both within the limit assigned.

**Table 4:** Comparison of NTM Elite agar unreadable plates and TVCs/*P. aeruginosa*.

	Brand	Type of Samples Collection	NTM ELITE Results	TVCs 22 °C CFU/mL	TVCs 36 °C CFU/mL	*P. aeruginosa* CFU/250 mL
1	Stockert 3T HCUs	pre-disinfection	non-AFB	>3 × 10^3^	>3 × 10^3^	<1
2	Stockert 3T HCUs	pre-disinfection	non-AFB	>3 × 10^3^	>3 × 10^3^	>150
3	Stockert 3T HCUs	post-disinfection	moulds	<1	<1	<1
4	Stockert 3T HCUs	pre-disinfection	non-AFB	>3 × 10^3^	>3 × 10^3^	>150
5	Stockert 3T HCUs	pre-disinfection	non-AFB	>3 × 10^3^	>3 × 10^3^	>150
6	Stockert 3T HCUs	pre-disinfection	non-AFB	>3 × 10^3^	>3 × 10^3^	>150
7	Stockert 3T HCUs	pre-disinfection	moulds	>3 × 10^3^	>3 × 10^3^	<1
8	Stockert 3T HCUs	pre-disinfection	non-AFB	>3 × 10^3^	>3 × 10^3^	<1
9	Stockert 3T HCUs	pre-disinfection	non-AFB	>3 × 10^3^	>3 × 10^3^	15

non-AFB: non-acid fast bacilli; TVC: total viable count; <1: not detected.

**Table 5:** Proficiency testing results (source: FEPTU report provided to laboratories that examine heater cooler unit (HCU) waters for *Mycobacterium* spp.)

Number of Distribution	Microorganism Strains by Each Distribution	CFU/100 mL
MY006 A	*M. chimaera*	14
	*(P. aeruginosa)*	(1.1 × 10^2^)
MY006 B	*M. chelonae*	2.2 × 10^2^
	*(B. multivorans)*	(1.5 × 10^2^)
MY008 A	*M. chelonae*	5.0 × 10^2^
	*(Microbacterium spp)*	(20)
	*(Staphylococcus xylosus)*	(42)
MY008 B	*M. chimaera*	8.0 × 10^2^
MY009 A	*M. chelonae*	5.8 × 10
	*(E. coli)*	(90)
MY009 B	*M. fortuitum*	30

## Data Availability

The dataset presented in the current study is not publicly available since data contains information that is being further used for other publication; however, it can be available to the editor upon request.
